# A quantitative *LumiFluo* assay to test inhibitory compounds blocking p53 degradation induced by human papillomavirus oncoprotein E6 in living cells

**DOI:** 10.1038/s41598-018-24470-4

**Published:** 2018-04-16

**Authors:** Lorenzo Messa, Marta Celegato, Chiara Bertagnin, Beatrice Mercorelli, Giulio Nannetti, Giorgio Palù, Arianna Loregian

**Affiliations:** Department of Molecular Medicine, University of Padua, Padua, Italy

## Abstract

High-risk human papillomaviruses (HR-HPVs) are the causative agents for the onset of several epithelial cancers in humans. The deregulated expression of the viral oncoproteins E6 and E7 is the driving force sustaining the progression of malignant transformation in pre-neoplastic lesions. Targeting the viral E6 oncoprotein through inhibitory compounds can counteract the survival of cancer cells due to the reactivation of p53-mediated pathways and represents an intriguing strategy to treat HPV-associated neoplasias. Here, we describe the development of a quantitative and easy-to-perform assay to monitor the E6-mediated degradation of p53 in living cells to be used for small-molecule testing. This assay allows to unbiasedly determine whether a compound can protect p53 from the E6-mediated degradation in cells, through a simple 3-step protocol. We validated the assay by testing two small molecules, SAHA and RITA, reported to impair the E6-mediated p53 degradation. Interestingly, we observed that only SAHA efficiently rescued p53, while RITA could not provide the same degree of protection. The possibility to specifically and quantitatively monitor the ability of a selected compound to rescue p53 in a cellular context through our *LumiFluo* assay could represent an important step towards the successful development of anti-HPV drugs.

## Introduction

Papillomaviruses (PVs) are small DNA viruses that can infect a wide range of mammals, including humans, and cause distinctive hyperproliferative lesions of the skin^1^. About 200 different viral genotypes are known to infect humans and a subset of these viruses, such as HPV16, HPV18, HPV31, HPV33, and HPV45, are classified as high-risk human papillomaviruses (HR-HPVs) due to their causative role in the development of several epithelial cancers, such as cervical, anogenital and some forms of oropharyngeal cancer^2^. An important clinical issue for the treatment of HPV-related diseases is the absence of specific anti-HPV drugs, and the development of a therapeutic vaccine remains an unmet medical need^3^. Thus, specific anti-HPV treatments are still globally required for the multitude of patients already suffering for HPV-induced cancers and for those already infected and at a high risk of developing HPV-associated carcinomas.

The ability of HPV to sustain epidermal neoplasias depends on the expression of the viral oncogenes *E6* and *E7*, which are the driving forces of keratinocyte hyperproliferation and, at a later stage, cancer progression^4^. E6 and E7 are small regulatory proteins important for efficient viral replication, which exert their functions through protein-protein interactions (PPIs) exclusively^5^. However, when the expression of *E6* and *E7* becomes unregulated, usually as a result of the integration of viral DNA into the host genome, their activities can efficiently induce malignant cell transformation by perturbing several signalling pathways involved in cell-cycle control, adhesion and differentiation^6^.

E6 is a very small cysteine-rich protein whose physiological role is to keep the infected cell in an S-phase-like state, cooperating with E7 for efficient cellular hijacking^7^. High-risk E6 proteins target p53 for proteasome-mediated degradation, while E7 can inhibit the activity of pRb, thus forcing the cell to continuously proliferate and accumulate somatic mutations^8^. E6 possesses a multifaceted inhibitory activity against p53, acting directly against the protein as well as against other cellular factors that normally lead to the activation of p53, such as p300 and ADA3^9–11^. In addition, E6 can bind several other cellular proteins to induce their degradation through the cellular proteasome machinery, such as procaspase 8, Bak, Scribble and MAGI-1^12–15^. The viral *E6* oncogene undergoes massive splicing events, producing several truncated isoforms in addition to the full-length protein, but only the latter mediates the degradation of p53^16–18^. Mechanistically, full-length high-risk E6 proteins can efficiently induce p53 degradation through the direct association with both p53 and the cellular ubiquitin ligase E6AP, to form a trimeric complex that leads to p53 ubiquitination and degradation^19^.

The intimate addiction of cancer cells to the sustained activity of E6 represents an advantage for the development of anti-cancer drugs, since perturbing E6 activities can restore the intracellular levels of active p53 and reactivate p53-mediated pathways, leading to oncogene-induced senescence and eventually apoptosis of cancer cells^20^. Many research groups have already addressed their attention to the development of an anti-E6 compound through different approaches^21–25^. Blocking the formation of the trimeric complex among E6, E6AP and p53 through a small-molecule compound represents a novel intriguing strategy to inhibit the E6-mediated degradation of p53 and to counteract the progression of HPV-associated cancers. Indeed, increasing successful examples of small-molecule PPI inhibitors, including candidate anticancer drugs, have been reported, thus highlighting the potential of targeting PPIs as a novel chemotherapeutic strategy^26–28^. However, for issues related to the structure, size and physico-chemical characteristics of E6, the development of a powerful anti-E6 compound interfering with crucial PPIs has proven to be a difficult task over the years. In addition, a major limit was represented by the lack of simple biological assays able to specifically and quantitatively evaluate the inhibitory activity of test compounds against the E6-mediated p53 degradation in a cellular context. To overcome this drawback, we developed a luminescence/fluorescence-based (*LumiFluo*) assay to quantitatively monitor the E6-mediated degradation of p53 in living cells to be used for small-molecule compound testing.

## Results

### Selection of the appropriate reporter system to monitor the E6-mediated degradation of p53

**Figure 1:**
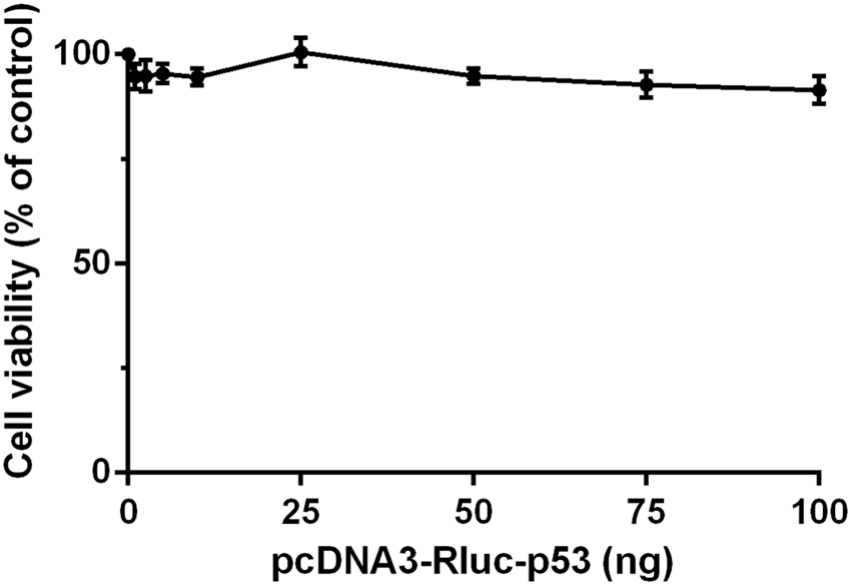
Effect of the expression of Rluc-p53 on the viability of H1299 cells. Cell viability was measured by the MTT assay following the transfection of increasing amounts of pcDNA3-Rluc-p53 plasmid into H1299 cells. Transfections were performed in 24-well plates and at 24 hours post-transfection cells were reseeded in a 96-well clear plate for viability determination. The control was represented by untransfected cells.

To measure the degradation of p53 by HR-HPV E6 oncoprotein, we chose H1299 cells as the candidate cell line for the purpose of this work. These cells appeared to be a suitable cellular reporter system for three reasons: (i) they are a p53^−/−^ cancer cell line wherein the expression of the endogenous p53 is abolished due to a homozygous deletion in the *TP53* gene; (ii) H1299 cells are epithelial cells devoid of any HPV sequence; and (iii) they are easily transfectable. To monitor the intracellular levels of the exogenous p53, cells were transiently transfected with pcDNA3-Rluc-p53 which encodes a fusion protein wherein *Renilla* luciferase (Rluc) is fused at the N-terminal end of p53. We first assessed the viability of transfected cells following the expression of Rluc-p53. H1299 cells were seeded in a 24-well plate, transfected with increasing amounts of pcDNA3-Rluc-p53 plasmid, or untransfected as a control, and reseeded in a 96-well plate. At 24 hours post-reseeding, cell viability was determined by the MTT assay. No significant reduction in the viability of transfected cells was observed independently from the amount of pcDNA3-Rluc-p53 used for transfection (Fig. 1). However, we observed changes in cell morphology from cobblestone-like to round-shaped cells in samples transfected with amounts of pcDNA3-Rluc-p53 higher than 50 ng (data not shown). Thus, we set this quantity as the threshold for pcDNA3-Rluc-p53 and subsequent transfections were scaled accordingly. In addition, in order to adapt our reporter system to direct luminometric measurements, in following experiments transfected cells were reseeded in 96-well white plates. To normalise for the number of cells plated upon reseeding and for transfection efficiency, cells were cotransfected with a plasmid expressing eGFP under the control of the cellular EF1α promoter and luminescent signals were normalised against fluorescence intensities. This approach allows to work with intact, living cells, avoiding the common step of cell lysis used for other normalisation procedures (e.g., normalisation with *Firefly* luciferase) since celenterazine (Rluc substrate) is a cell-permeable molecule and eGFP only requires excitation.

### Effect of cell density on the emission of Rluc-p53

**Figure 2:**
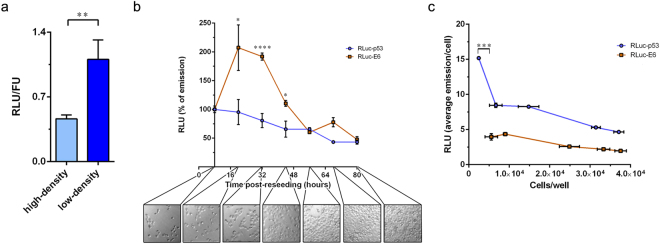
Cell density-dependent regulation of Rluc-p53. (**a**) Different accumulation of Rluc-p53 within H1299 cells cotransfected with pcDNA3-Rluc-p53 and pcDNA3.1-EF-1α-GFP and reseeded in a 96-well white plate at high density (3 × 10^4^ cells/well) or low density (3 × 10^3^ cells/well) (**p < 0.01). (**b**) H1299 cells were transfected with pcDNA3-Rluc-p53 or pcDNA3-Rluc-E6, then the next day cells were reseeded at low density (3 × 10^3^ cells/well) in 96-well white plates. Rluc emissions were monitored over the course of 72 hours post-reseeding, until cells reached confluence. The first measurement was performed at 8 hours post-reseeding in 96-well white plates and was set as 100%. Subsequent measurements were performed every 12 hours. A representative picture of the cell monolayer at each time point is shown (*p < 0.05, ****p < 0.0001 of cells transfected with pcDNA3-Rluc-E6 versus pcDNA3-Rluc-p53-transfected samples). **(c)** H1299 cells were transfected as in (**b**) and reseeded in 96-well white plates at different densities. At 24 hours post-reseeding, Rluc emissions were measured and cells were subsequently trypsinised and counted in duplicate (***p < 0.001).

Several studies have reported the influence of cell density on the steady-state levels of p53^29–31^. High numbers of cell-cell contacts, which recapitulate the high-density cell culture conditions, were shown to down-regulate the intracellular levels of p53, likely in an Mdm2-dependent manner^32^. Indeed, when transiently transfected H1299 cells were reseeded at low density in 96-well white plates, the relative emission of Rluc-p53 was substantially higher when compared to its emission in dense culture (Fig. 2a). In addition, we monitored the emission of Rluc-p53 over the course of 72 hours post-reseeding in 96-well white plates. As a control, we transiently transfected H1299 cells with a recombinant construct expressing *Renilla* luciferase fused at the N-terminal end of HPV16 E6 (Rluc-E6) in order to compare the expression and intracellular accumulation of the exogenous p53 and E6 proteins by means of the time-dependent emission of the Rluc moiety. Strikingly, while the emission levels of Rluc-E6 were substantially higher after 24 hours (which roughly correspond to 16 hours of active cell proliferation), as justified by the increase in cell number, the emission of Rluc-p53 never increased but rather progressively decreased over the course of time, even during the initial phase of active cell proliferation (i.e., within the initial 32 hours post-reseeding) (Fig. 2b). The absence of a peak in the time-dependent emission curve of Rluc-p53 suggested that the highest intracellular level of Rluc-p53 is maintained only during the first 20–24 hours post-reseeding (approximately corresponding to 12–16 hours of active cell proliferation), i.e., when the number of cell-cell contacts is very low. Indeed, when transiently transfected H1299 cells were plated at different densities, the highest average emission of Rluc-p53 per cell was detected in sparse cultures, only when the cells were spatially separated from each other (Fig. 2c). On the contrary, the average emission per cell of Rluc-E6 was unaffected by the number of cell-cell contacts and slightly decreased only in very dense culture conditions, when cells are characterised by low protein synthesis and metabolism. Importantly, our results agree with a previous report indicating that the highest intracellular accumulation of ectopically expressed p53 in H1299 cells is achieved in low-density cell culture conditions^32^.

### HPV16 E6 efficiently promotes Rluc-p53 degradation

**Figure 3:**
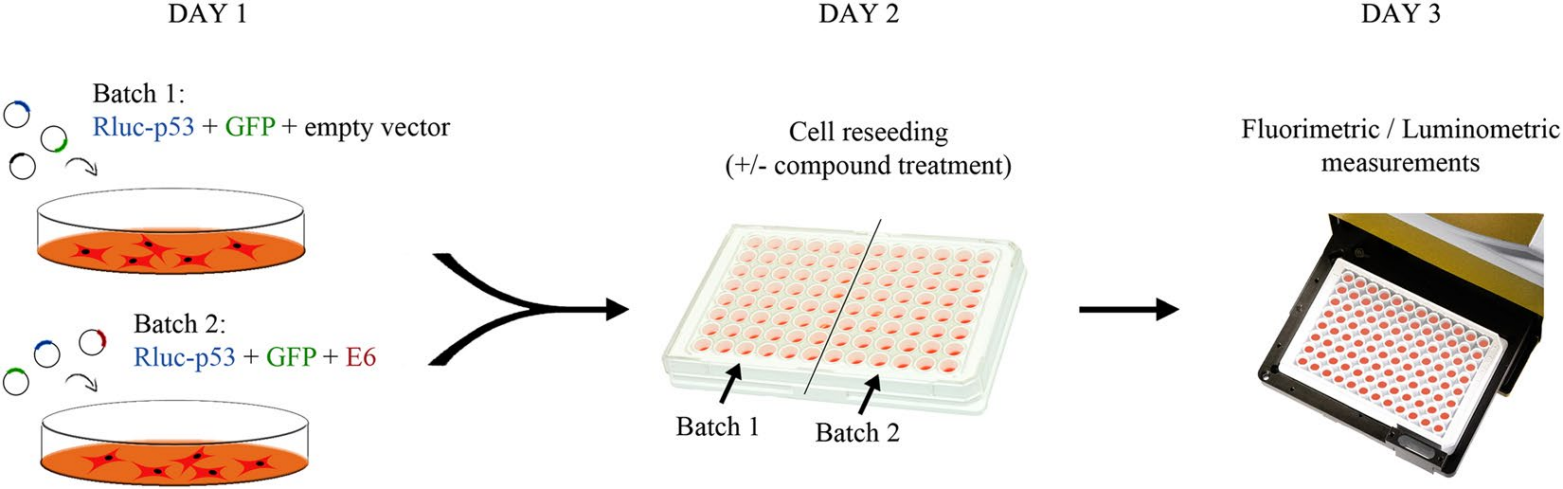
Schematic representation of the experimental procedure. The first day, two batches of H1299 cells are concomitantly transfected with plasmids encoding Rluc-p53 and eGFP along with a plasmid encoding HPV16 E6 or with empty vector. The second day, cells from both batches are reseeded in 96-well white plates at low density (3 × 10^3^ cells/well) as described in the text. In experiments testing candidate inhibitors, at 8 hours post-reseeding test compounds are added to both Batch 1 and Batch 2 samples. At day three, fluorimetric and luminometric measurements are performed. The RLU/FU ratio of cells cotransfected with empty vector (Batch 1) is set as 100% and related to the RLU/FU ratio of cells cotransfected with the E6-expressing plasmid (Batch 2) in order to calculate the ratio of Rluc-p53 degradation mediated by E6.

**Figure 4:**
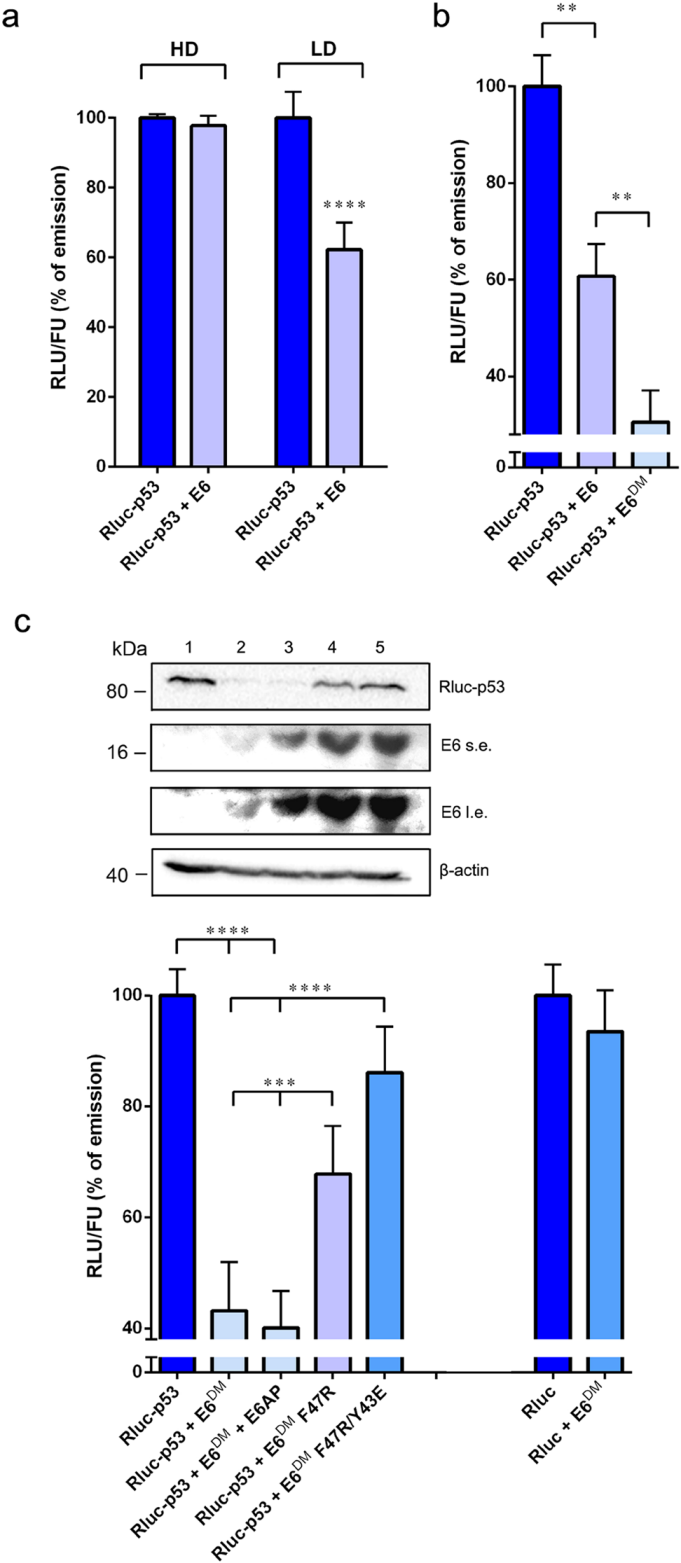
Rluc-p53 is actively degraded by full-length HPV16 E6 in low-density cell culture conditions. (**a**) H1299 cells were cotransfected with pcDNA3-Rluc-p53 and pcDNA3.1-EF-1α-GFP plasmids along with p513-E6 or with p513 empty vector and reseeded in a 96-well white plate at high density (HD) or low density (LD) for fluorimetric and luminometric analysis (****p < 0.0001). (**b**) H1299 cells were transfected as in (**a**) in the presence of p513-E6, p513-E6^DM^ or p513 empty vector and reseeded at low density in a 96-well white plate (**p < 0.01). (**c**) Upper panel: Western blot analysis of H1299 cells cotransfected with pcDNA3.1-EF-1α-GFP and pcDNA3-Rluc-p53 plasmids in the presence of p513 empty vector (1), p513-E6^DM^ (2), p513-E6^DM^ and pcDNA3-E6AP (3), p513-E6^DM^ F47R (4) or p513-E6^DM^ F47R/Y43E (5). β-actin was used as a loading control; s.e., short exposure; l.e., long exposure. Lower panel: relative RLU/FU ratios of H1299 cells transfected as described in the upper panel (from 1 to 5) and reseeded at low density in a 96-well white plate. As a specificity control, H1299 cells were cotransfected with pcDNA3.1-EF-1α-GFP and pcDNA3-Rluc plasmids along with p513-E6^DM^ or with p513 empty vector (right columns). The RLU/FU ratios of cells cotransfected with empty vector were set as 100% in every experiment (***p < 0.001, ****p < 0.0001).

In order to detect the E6-mediated degradation of p53 in a cellular context, we performed the assay by cotransfecting H1299 cells with a HPV16 E6-expressing plasmid. High-risk E6 oncoproteins mediate the ubiquitination and degradation of p53 through the formation of a heterotrimeric complex that involves the direct binding between E6, p53 and the cellular ubiquitin ligase E6AP^19^. In order to avoid any steric hindrance caused by the addition of tags, which could result in an impairment of p53 degradation, untagged HPV16 E6 was chosen for the purpose of this assay. To monitor the relative decrease in the emission of Rluc-p53, as a result of its degradation mediated by the viral E6 oncoprotein, two batches of H1299 cells were transfected in parallel with pcDNA3.1-EF-1α-GFP and pcDNA3-Rluc-p53 along with either p513-E6 or p513 empty vector, as schematically represented in Fig. 3. After overnight incubation, transfected cells were trypsinised, counted and reseeded in 96-well white plates. Initially, two cell densities were tested, i.e., 3 × 10^3^ cells/well (low-density condition) and 3 × 10^4^ cells/well (high-density condition). The next day, luminescent signals were measured and normalised against the fluorescence of eGFP. The emission signals of cells cotransfected with p513-E6 were then compared to those of cells cotransfected with empty vector. Indeed, the expression of wild-type HPV16 E6 reduced the emission of Rluc-p53, although not dramatically, but only in H1299 cells reseeded at low density (Fig. 4a). Thus, in keeping with our findings on the density-dependent regulation of p53 levels (Fig. 2), these results indicated that the degradation of ectopically expressed p53 mediated by HR-HPV E6 oncoprotein in H1299 cells can be efficiently detected only in low-density cell culture conditions.

High-risk E6 oncoproteins are translated from a bicistronic mRNA that produces a pool of different proteins, including E7 and several truncated isoforms of E6 (E6*I, E6*II, E6^E7) resulting from mRNA splicing^16^. Several studies showed the inability of these truncated isoforms to efficiently induce the degradation of p53, but their expression levels, in particular that of E6*I, equal that of the full-length protein, limiting the degradation activity of E6 through direct binding and modulation^17,18^. Indeed, when we introduced a silent point mutation in the splicing donor site of the *E6* gene (E6^DM^), which abrogates the expression of the smaller isoforms in favour of the full-length protein, the decrease in the emission of Rluc-p53 observed upon coexpression with E6 was markedly enhanced in H1299 cells plated at low density (Fig. 4b).

Next, to test the specificity of the assay, we assayed mutants of HPV16 E6 (E6^DM^ F47R and E6^DM^ F47R/Y43E) that were reported to possess a reduced ability to induce the degradation of p53 due to one or two amino acid substitutions in the p53-binding interface of E6^19,33^. As expected, the ability of E6^DM^ F47R and E6^DM^ F47R/Y43E to induce a decrease in the emission of Rluc-p53 was significantly impaired as compared to the wild-type full-length E6^DM^ protein (Fig. 4c, lower panel). These results were confirmed by Western blot analysis, as we observed a strong reduction of the intracellular levels of Rluc-p53 in H1299 cells cotransfected with p513-E6^DM^ vector, while E6^DM^ F47R and E6^DM^ F47R/Y43E could not significantly degrade Rluc-p53 (Fig. 4c, upper panel), in agreement with Rluc measurements. Furthermore, we tested whether the transient cotransfection of a plasmid expressing E6AP could further enhance the degradation of Rluc-p53 induced by E6^DM^. Interestingly, the cotransfection of pcDNA3-E6AP did not increase the rate of degradation of Rluc-p53, suggesting that the overexpression of E6AP is dispensable for the productive degradation of Rluc-p53 in this experimental setting (Fig. 4c, lower panel). The activity of E6^DM^ was also tested against Rluc, as a way to determine the specificity of the degradation activity towards the p53 moiety rather than the tagged luciferase. As expected, no significant reduction in the emission of Rluc was detected (Fig. 4c).

Finally, in order to demonstrate the feasibility of using this assay for high-throughput screenings, we calculated the Z′-factor. To this end, we concomitantly cotransfected three batches of H1299 cells with plasmids encoding Rluc-p53 and eGFP along with p513-E6^DM^, p513-E6^DM^ F47R/Y43E, or p513 empty vector, respectively. Transfected cells were then re-seeded in 96-well white plates and the assay was performed as described above. The population of cells cotransfected with the p513-E6^DM^ plasmid represented the negative control (highest rate of Rluc-p53 degradation), while cells cotransfected with the p513-E6^DM^ F47R/Y43E plasmid represented the positive controls (lowest rate of Rluc-p53 degradation). We calculated a Z′-factor of 0.71, thus demonstrating an overall good reproducibility and the possibility to employ this assay for high-throughput screening campaigns.

### Assay validation using known anti-cervical cancer drugs

**Figure 5:**
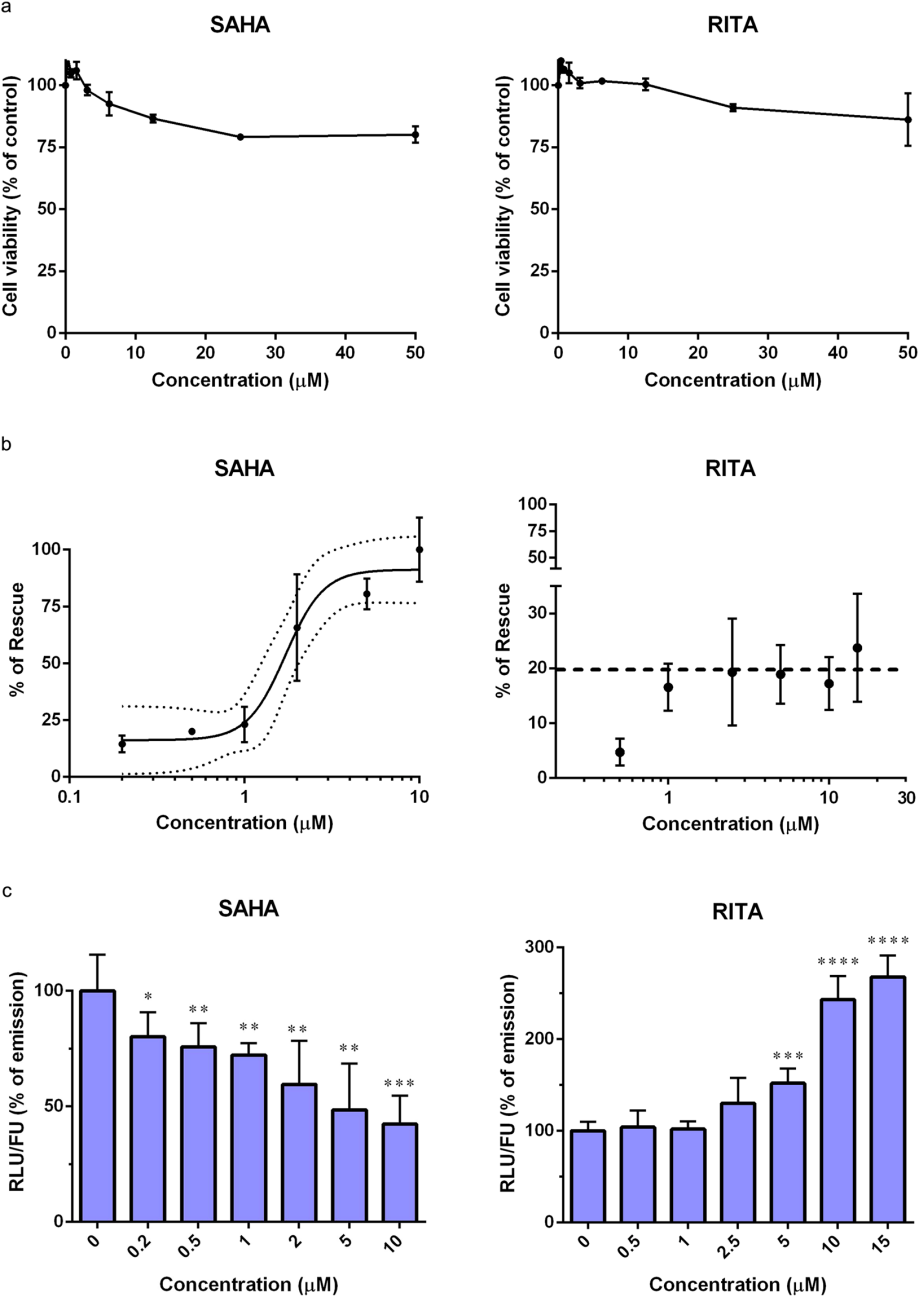
SAHA, but not RITA, efficiently rescues Rluc-p53 from the degradation induced by HPV16 E6. (**a**) Cytotoxic effects of SAHA and RITA in untransfected H1299 cells. The control was represented by untreated cells. (**b**) Dose-response analysis of the rescue of Rluc-p53 in transfected H1299 cells following treatment with increasing concentrations of SAHA and RITA for 24 hours. The black line in the graph of SAHA-mediated rescue of Rluc-p53 represents the sigmoidal Four Parameter Logistic (4PL) regression curve while dotted lines indicate the 95% confidence interval of the model. The dashed line in the graph of RITA-mediated rescue of Rluc-p53 indicates the maximum average rescue obtained after treatment with concentrations of RITA ≥ 2.5 μM. (**c**) Dose-dependent effect of SAHA and RITA on the intracellular levels of Rluc-p53 in H1299 cells cotransfected with p513 empty vector. The average RLU of DMSO-treated cells (no compound) was set as 100% in every experiment (*p < 0.05, **p < 0.01, ***p < 0.001, ****p < 0.0001 of compound-treated samples versus the DMSO-treated control).

In order to demonstrate the ability of the *LumiFluo* assay to identify compounds capable of protecting p53 from the degradation mediated by E6, we selected two small molecules that were shown to impair the E6-mediated degradation of p53 in a cellular context, namely Vorinostat (SAHA) and the p53-targeting compound RITA. These two small molecules were reported to rescue p53 levels in cervical cancer cells, albeit through different molecular mechanisms^34–36^. Prior to dose-response analyses, in order to investigate whether SAHA and RITA might affect cell viability in a p53-independent manner, we assessed the cytotoxic effects of these two compounds in untransfected H1299 cells by the MTT assay. Both SAHA and RITA did not significantly affect the viability of H1299 cells at any concentration tested up to 50 μM (Fig. 5a). Then, H1299 cells were transiently cotransfected with plasmids encoding Rluc-p53 and eGFP along with p513-E6^DM^ or p513 empty vector as previously described and treated with SAHA and RITA for 24 hours. In order to account for any possible off-target effect induced by compound treatment that might affect the intracellular levels of Rluc-p53 (e.g., general upregulation/downregulation of transcription, translation or cell metabolism), we treated both H1299 cells cotransfected with p513-E6^DM^ and cells cotransfected with p513 empty vector. The RLU/FU ratio of compound-treated E6-expressing cells was then compared against that of cells not expressing E6^DM^ treated with the same compound concentration, as a way to specifically measure the rescue of Rluc-p53 from the degradation activity of E6. The rate of degradation of Rluc-p53 mediated by E6 measured in treated cells (Δ^trd^) was then compared to the rate of degradation measured in untreated controls (Δ^unt^). To quantitatively measure the rescue of Rluc-p53 induced by SAHA and RITA, a dose-response analysis was performed over a wide range of concentrations to obtain compounds’ IC_50_ values. Taking into account the available data reported in the literature^34,36^, SAHA was tested in a range of concentrations from 0.2 μM to 10 μM, and RITA from 0.5 μM to 15 μM. Interestingly, SAHA successfully inhibited the E6-mediated p53 degradation with an IC_50_ value of 1.79 ± 0.50 μM (Fig. 5b), without affecting the intracellular accumulation of neither E6, nor of E6AP (Supplementary Fig. 1). Unexpectedly, RITA was unable to rescue Rluc-p53 from the degradation induced by E6 in a dose-dependent manner (Fig. 5b). In particular, we observed that treatment with RITA at concentrations ≤2.5 μM induced a dose-dependent rescue of Rluc-p53 as previously reported^34^, albeit weakly. However, when cells were treated with RITA at concentrations above 2.5 μM, the rescue of Rluc-p53 settled at a constant value of approximately 20%, suggesting that RITA could not efficiently protect Rluc-p53 from the E6-mediated degradation in a dose-dependent manner, preventing the possibility to estimate an IC_50_ value by this assay (Fig. 5b). Nevertheless, we observed that RITA treatment in H1299 cells cotransfected with p513 empty vector induced a dose-dependent increase of the intracellular levels of Rluc-p53, indicating that RITA can indeed stabilise and upregulate p53 in the absence of E6 (Fig. 5c). Conversely, we observed that treatment with SAHA in H1299 cells cotransfected with p513 empty vector induced a dose-dependent decrease of the intracellular levels of Rluc-p53, in line with previous reports indicating that SAHA generally affects translation, reducing protein synthesis^37–39^.

## Discussion

HPV-associated cancers possess the unique characteristic of being intimately addicted to the continuous activity of the major viral oncoproteins E6 and E7, and unlike many other solid tumors, they retain wild-type p53 and pRb^40^. Impairing the functions of E6 through an inhibitory small-molecule compound could have profound effects on the survival of cancer cells due to the reactivation of p53-mediated pathways and thus represents a fascinating strategy for the treatment of HPV-associated neoplasias^41,42^. However, the successful development of anti-HPV drugs has been so far hampered also by the lack of specific and quantitative bioassays capable of assessing the inhibitory activity of candidate compounds against E6 functions in a cellular context. Indeed, the molecular assays so far available in the field of anti-HPV drug discovery were mostly restricted to *in vitro* experiments, such ELISAs, pull-down assays, and p53 degradation assays using *in vitro* translated p53 protein, which are all cell-free systems and do not allow any reliable prediction on the activity of test compounds in the cell^23,24,43,44^. Indeed, such an experimental approach often leads to false positive results, i.e., compounds active *in vitro* that are less active or even completely inactive in cell-based systems. Previously, the monitoring of compound-induced inhibition of the E6-mediated degradation of p53 in cells was almost exclusively performed through western blotting, detecting the eventual accumulation of p53 in HPV-positive cells (i.e. HeLa, CaSki, and SiHa) following cell treatment, harvest, lysis and analysis of cellular lysates. Undoubtedly, this approach is poorly feasible during the initial phases of drug discovery, when a high number of compounds have to be tested. In addition, the eventual upregulation of the endogenous p53 in HPV-positive cells could be a result of indirect mechanisms and off-target effects induced by compound treatment, such as an induction of p53 due to genotoxic stress or general changes in gene transcription and translation, rather than a specific direct inhibition of the E6-mediated p53 degradation. Although Mesplède and colleagues previously reported a similar strategy to monitor the degradation of p53 by E6 proteins of different HPV genotypes^45,46^, they never used such an approach for testing inhibitory compounds. Thus, taking advantage of a pre-existing experimental approach, we report the development and validation of a luminescence/fluorescence-based (*LumiFluo*) cellular assay that specifically monitors the HPV E6-mediated degradation of p53 in living cells and can be employed to quantitatively test the ability of candidate small molecules to protect p53 from the degradation activity of E6.

In the assay development, the absence of endogenous p53 represented a major feature in the choice of the cell line to be used, as well as the absence of endogenously expressed HPV genes; thus, we chose the H1299 cell line for the purpose of this assay. Conversely, other groups have preferred the use of C33A cells instead of H1299 cells to monitor the E6-mediated Rluc-p53 degradation^45–47^. However, C33A cells express high levels of endogenous p53 due to an Arg to Cys amino acid substitution at position 273 which makes the protein inactive and highly expressed. Such a high intracellular accumulation could represent a drawback for the purpose of this assay, since the endogenous mutated p53 of C33A cells was shown to be still efficiently bound and degraded by E6^33^, thus possibly introducing a bias in the determination of compounds’ IC_50_ values in the presence of ectopically expressed Rluc-p53. Noteworthy, control experiments showed that the ectopic expression of HPV16 E6 in C33A cells resulted in the concomitant degradation of both endogenous p53 and ectopically expressed Rluc-p53 (Supplementary Fig. 2). These results also indicated that the experimental approach of this assay indeed reflects the biological context of HPV-induced tumours, wherein the endogenous expression of E6 induces the dramatic reduction of p53 levels.

A major innovative feature of this assay is the correlation between the density-dependent regulation of p53 and its degradation mediated by E6, which is hardly detectable if the experiment is performed in high-density cell culture conditions (Fig. 4a). To our knowledge, this was never addressed in any study dealing with anti-HPV drug discovery before and uncovers a new important methodological aspect for the successful development of anti-E6 compounds. Interestingly, C33A cells tend to massively detach from the surface when kept in culture, and thus we speculate this might be the reason why others have preferred the use of those cells to monitor the E6-mediated degradation of p53, since C33A cells hardly reach confluence.

A third important feature of our *LumiFluo* assay is the possibility to finely tune the selection of small molecules that directly impair the degradation of p53 induced by E6, because off-target effects are subtracted during data analysis. Indeed, off-target effects can represent a major issue in the field of drug discovery, particularly when using cell-based assays, because selected compounds can induce undesired effects through unpredicted molecular mechanisms (such as general nonspecific effects on cellular transcription, metabolism, protein synthesis and turnover), thus introducing a bias. In our experimental setting we can avoid such biases by concomitantly treating both cells cotransfected with the E6-encoding plasmid and cells cotransfected with empty vector and then calculating the rate of Rluc-p53 degradation in treated samples to be related to the rate of Rluc-p53 degradation in untreated controls. This approach strengthens the potency of this assay to unbiasedly determine the ability of a compound to specifically protect p53 from the E6-mediated degradation independently of other indirect effects that might impact on the intracellular accumulation of Rluc-p53.

In order to validate the ability of this assay to specifically identify compounds capable of impairing the E6-mediated degradation of p53, we tested two small molecules (SAHA and RITA) that were reported to rescue p53 from the degradation induced by E6 in cervical cancer cells. SAHA is an FDA-approved histone deacetylase inhibitor (HDACi) that was shown to impair p53 degradation and downregulate E6 and E7 protein levels in cervical cancer cells^36,48^. Its activity leads to an increase in p53 acetylation, a mechanism known to stabilise and enhance p53 transactivation activity, resulting in the accumulation of its intracellular levels^35^. RITA is a p53 agonist compound that was shown to directly bind p53, inducing a conformational change that prevents its interaction with Mdm2 and E6AP, the latter of which is indispensable for the degradation of p53 mediated by E6^34,49^. However, an increasing body of evidence successively suggested that the main mechanism of action of RITA may be the induction of DNA damage that leads to the accumulation of p53 levels, rather than a direct binding of RITA to p53^50,51^. By applying our assay, we confirmed that SAHA directly protects p53 from the degradation induced by HPV16 E6 in a dose-dependent manner, while RITA is unable to provide a dose-dependent rescue of p53 at concentrations higher than 2.5 μM (Fig. 5b). Importantly, our results are in agreement with previous reports indicating that hyperacetylated p53, which accumulates following treatment with a HDACi, can no longer be degraded by E6^48^, while whether RITA binds to p53 is still debated^51,52^. Nevertheless, we argue that the partial rescue of p53 induced by RITA could recapitulate the effect observed by Zhao and colleagues in HPV-positive cancer cells^34^. Furthermore, we observed that the IC_50_ value that we determined for SAHA (1.79 ± 0.50 μM) is very close to the concentration that He and colleagues determined to inhibit HDAC activity in HeLa cells (2.5 μM)^36^. Finally, the possibility to analyse the effects of compound treatment on the intracellular levels of Rluc-p53 in the absence of E6 could additionally serve as a way to determine whether a compound can influence the intracellular accumulation of Rluc-p53. Indeed, we observed that SAHA downregulates the intracellular levels of Rluc-p53 in a dose-dependent manner, an effect that can be ascribed to the previously reported propensity of SAHA to generally impair protein synthesis^37–39^. RITA, instead, was capable of inducing a dose-dependent increase of Rluc-p53 intracellular levels, up to a 2.5-fold increase when cells were exposed to a RITA concentration of 15 μM (Fig. 5c). This observation is in line with the notion that RITA activity converges on p53 accumulation and transactivation^49^, albeit our results suggest that RITA is unable to provide a direct specific protection from the degradation mediated by E6.

In conclusion, we developed a simple, sensitive, and quantitative cell-based assay that measures the degradation of p53 mediated by the HR-HPV E6 oncoprotein in living cells to be used for small-molecule compound testing. Most importantly, by this assay we can specifically determine the ability of a small molecule to inhibit the E6-mediated degradation of p53 in a cellular context. Although we tested only two compounds for assay validation, our assay could be easily scaled up to test higher numbers of compounds and possibly also amenable to automation. In addition, the approach described proposes a cell-based strategy that could be potentially also exploited for the identification of compounds that act, through different mechanisms, as general p53 agonists. Thus, our *LumiFluo* assay can represent a new useful biological tool in the field of anti-HPV drug discovery, serving as a platform for the development of new therapeutic agents for the treatment of HPV-associated neoplasias.

## Methods

### Plasmid construction and mutagenesis

pcDNA3-Rluc-p53 and pcDNA3-Rluc-E6 plasmids were generated by the Gateway® cloning system (Thermo Fisher Scientific). Genes encoding wild-type human p53 and HPV16 E6 were cloned into a Gateway® entry vector (pDONR207) using BP Clonase (Thermo Fisher Scientific) according to manufacturer’s protocol. Sequence-verified clones were used to transfer p53 and HPV16 E6 sequences into a Rluc-expressing Gateway® destination vector using LR Clonase (Thermo Fisher Scientific) according to manufacturer’s protocol. Final constructs consisted of full-length wild-type p53 or E6 fused at the C-terminal end of Rluc. The p513-E6 and pcDNA3-E6AP plasmids were a kind gift of Dr. Lawrence Banks (ICGEB, Trieste, Italy). The p513 empty vector was constructed by removing the *E6* sequence with BamHI and NotI restriction enzymes and subsequently filling and removing 5′ and 3′ overhangs, respectively, using Klenow DNA Polymerase (NE BioLabs) for blunt-end ligation. Mutations in the HPV16 *E6* gene were introduced by the QuikChange® Site-Directed mutagenesis system (Stratagene) according to manufacturer’s protocol. The HPV16 E6 splice donor site was mutated from GAGGTA to GAAGTA to create the splicing-defective E6 mutant (E6^DM^), as previously described^53^. The p53 degradation-defective E6 mutants were created by substituting Phe47 and Tyr43 into Arg and Glu, respectively, as reported by Zanier *et al*.^33^. pcDNA3.1-EF-1α-GFP was previously reported^54^.

### Cell culture and transfection

The non-small-cell lung carcinoma H1299 cell line was grown in Dulbecco’s modified Eagle medium (DMEM, Thermo Fisher Scientific), supplemented with 10% foetal bovine serum and 1% penicillin/streptomycin (Thermo Fisher Scientific). Transfections were performed with Lipofectamine 3000 (Thermo Fisher Scientific) according to manufacturer’s protocol with a constant Lipofectamine-DNA ratio of 2:1 (µl/µg).

### Chemical compounds

RITA was purchased from Cayman Chemical, while SAHA from Sigma-Aldrich. Both compounds were dissolved in DMSO and stock solutions were stored at −20 °C.

### Cell viability assay

Cell viability was determined by the 3-(4,5-dimethylthiazol-2-yl)-2,5-diphenyl tetrazolium bromide (MTT) method, as previously reported^55^. To evaluate the viability of H1299 cells transfected with pcDNA3-Rluc-p53, cells were reseeded in quadruplicate in a 96-well clear plate and cell viability was measured at 24 hours post-reseeding. To evaluate compounds cytotoxicity, 3 × 10^3^ H1299 cells were seeded in a 96-well clear plate and treated the next day with scalar dilutions of test compounds in complete medium. Cell viability was then measured 24 hours post-treatment.

### Western Blot analysis

At 24 hours post-transfection, H1299 cells were collected and lysed in RIPA buffer (50 mM Tris, 150 mM NaCl, 1% NP-40, 0.1% SDS) supplemented with Halt Protease Inhibitor Cocktail (Thermo Fisher Scientific). Cellular lysates were separated by SDS-PAGE, transferred onto polyvinylidene difluoride (PVDF) membranes and analysed by Western blotting. Anti-p53 (DO-1, 1:4,000), anti-E6AP (E-4, 1:500) and anti-HPV16 E6 (C-19, 1:100) primary antibodies were from Santa Cruz, while anti-β-actin (A5441, 1:8,000) was from Sigma-Aldrich. Horseradish peroxidase-conjugated goat anti-mouse (sc-2055, 1:2,000) and rabbit anti-goat (AP106P, 1:2,000) secondary antibodies were from Santa Cruz and Millipore, respectively.

### *LumiFluo* assay and data analysis

Approximately 1.5 × 10^5^ H1299 cells were seeded in a 6-well plate and after at least 8 hours to allow proper cell attachment, were transfected as described above. Two batches of cells were concomitantly cotransfected with pcDNA3.1-EF-1α-GFP and pcDNA3-Rluc-p53 along with either the E6-expressing plasmid or p513 empty vector with a constant eGFP/Rluc-p53/E6 ratio of 10:2:1. The next day, transfected cells were trypsinised, counted and reseeded in quadruplicate at 3 × 10^3^ cells/well (low-density condition) in 96-well white plates using phenol red-free medium (Thermo Fisher Scientific). After at least 8 hours post-reseeding to allow proper cell attachment, test compounds were added in complete phenol red-free medium both to cells cotransfected with the p513-E6^DM^ plasmid and to cells cotransfected with p513 empty vector. Controls were represented by cells cotransfected with pcDNA3.1-EF-1α-GFP and pcDNA3-Rluc-p53 along with the E6-expressing plasmid or empty vector treated with DMSO at the same concentration of compound-treated cells. At 24 hours post-treatment, data collection was performed by first measuring eGFP emission and subsequently Rluc luminescence. Cells were washed with phosphate buffered saline (PBS) and exposed at an excitation wavelength of 485 nm, measuring eGFP emission at 535 nm, integrated over 0.1 s using a VICTOR X2 Multilabel Plate Reader (PerkinElmer). Luminometric analysis was performed by removing PBS and adding 30 µl/well of benzyl-coelenterazine (P.j.k. GmbH) at a final concentration of 5 µM in PBS, measuring the emission signal at 460 nm, integrated over 2 s at 3 minutes after substrate addition. The rate of Rluc-p53 degradation was calculated as follows:

1 $${\rm{\Delta }}=({(RLU/FU)}^{\mathrm{E6}-}\mbox{--}{(\mathrm{RLU}/\mathrm{FU})}^{{\rm{E6}}+})/{(\mathrm{RLU}/\mathrm{FU})}^{\mathrm{E6}-}$$

where (RLU/FU)^E6−^ represents the average luminescent signal, normalised over the fluorescence of eGFP, of cells cotransfected with the p513 empty vector, and (RLU/FU)^E6+^ represents the average luminescent signal, normalised over the fluorescence of eGFP, of E6-expressing cells. To calculate the percentage of rescue (R) of Rluc-p53 upon compound treatment, the rate of Rluc-p53 degradation in treated cells (Δ^trd^) was related to the rate of degradation in control cells treated with DMSO (Δ^unt^) as follows:

2 $$R=(1-({{\rm{\Delta }}}^{{\rm{trd}}}/{{\rm{\Delta }}}^{{\rm{unt}}}))\ast 100$$

### Statistical analysis

Data were analysed with unpaired Student *t* test or one-way analysis of variance (ANOVA) and IC_50_ values were calculated by four parameter logistic (4PL) non-linear regression using GraphPad Prism 6 (GraphPad Software Inc.). Values were presented as mean ± SEM of at least three independent experiments. Statistical significance was calculated with a *p* value < 0.05. To test assay reproducibility, we performed the *LumiFluo* assay with 48 replicates of positive and negative controls (24 for each control group), with each replicate composed of a triplicate of cells seeded in 96-well white plates. Z′-factor was calculated using the equation previously described by Zhang and colleagues^56^.

### Data availability

All data generated or analysed during this study are included in this published article.

## Electronic supplementary material

Supplementary Information
