# Supplementary material for: A quantitative LumiFluo assay to test inhibitory compounds blocking p53 degradation induced by human papillomavirus oncoprotein E6 in living cells
Source: Sci Rep. 2018 Apr 16;8:6020. doi: 10.1038/s41598-018-24470-4 (PMC5902497; doi:10.1038/s41598-018-24470-4)
Supplement: Supplementary file 1 — Supplementary Information [file 41598_2018_24470_MOESM1_ESM.pdf]

A quantitative *LumiFluo* assay to test inhibitory compounds blocking p53 degradation induced by human papillomavirus oncoprotein E6 in living cells

Lorenzo Messa, Marta Celegato, Chiara Bertagnin, Beatrice Mercorelli, Giulio Nannetti, Giorgio Palù & Arianna Loregian

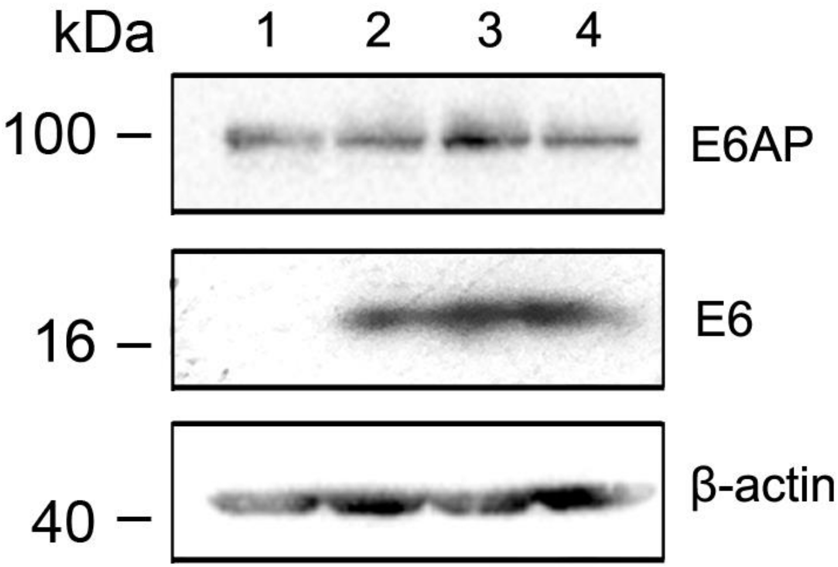

Supplementary Figure 1. Effect of SAHA treatment on the intracellular levels of E6 and E6AP. Western blot analysis of H1299 cells cotransfected with pcDNA3.1-EF-1α-GFP and pcDNA3-Rluc-p53 plasmids along with p513 empty vector (1) or p513-$E6^{DM}$ (2-4). Transfected cells were treated with DMSO (1-2), SAHA 2 µM (3), or SAHA 5 µM (4) for 24 hours. β-actin was used as a loading control.

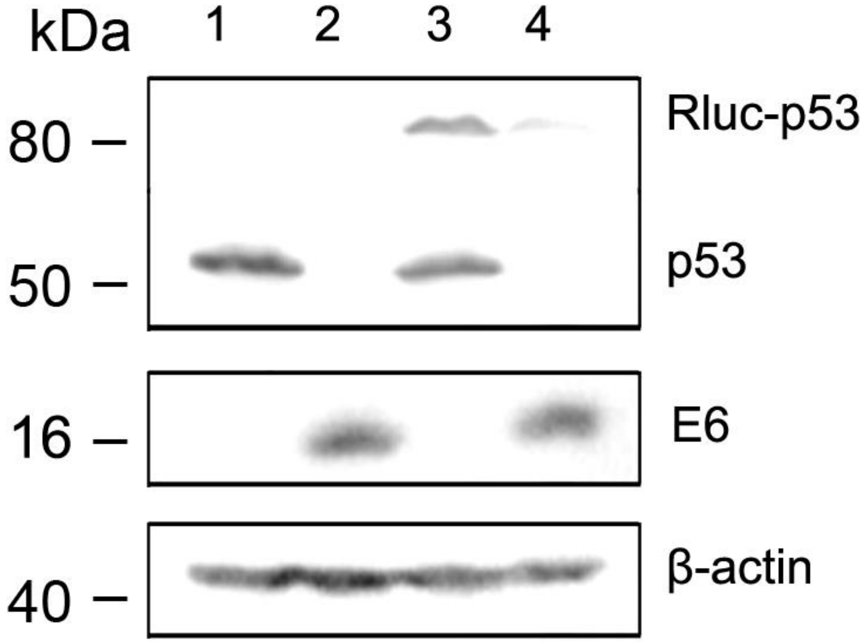

Supplementary Figure 2. HPV16 E6 can concomitantly target endogenous p53 and ectopically expressed Rluc-p53 to degradation. Western blot analysis of C33A cells transfected with p513 empty vector (1), p513-E6^{DM} (2), pcDNA3-Rluc-p53 (3) or cotransfected with p513-E6^{DM} and pcDNA3-Rluc-p53 (4). β-actin was used as a loading control.
